# ﻿Symbiotic synergy: How Arbuscular Mycorrhizal Fungi enhance nutrient uptake, stress tolerance, and soil health through molecular mechanisms and hormonal regulation

**DOI:** 10.3897/imafungus.16.144989

**Published:** 2025-03-21

**Authors:** Nazir Ahmed, Juan Li, Yongquan Li, Lifang Deng, Lansheng Deng, Muzafaruddin Chachar, Zaid Chachar, Sadaruddin Chachar, Faisal Hayat, Ahmed Raza, Javed Hussain Umrani, Lin Gong, Panfeng Tu

**Affiliations:** 1 College of Horticulture and Landscape Architecture, Zhongkai University of Agriculture and Engineering, Guangdong, 510550, Guangzhou, China; 2 Institute of Biomass Engineering, South China Agricultural University, 510642, Guangzhou, China; 3 College of Natural Resources and Environment, South China Agricultural University, 510642, Guangzhou, China; 4 Faculty of Crop Production, Sindh Agriculture University, 70060), Tandojam, Pakistan; 5 College of Agriculture and Biology, Zhongkai University of Agriculture and Engineering, Guangdong, 510550, Guangzhou, China; 6 Department of Integrative Agriculture, College of Agriculture and Veterinary Medicine, United Arab Emirates University, Al Ain, United Arab Emirates; 7 Dongguan Yixiang Liquid Fertilizer Co. Ltd, 523135, Dongguan, China

**Keywords:** AM inoculation, hormonal signaling, metal sequestrations, plant mineral nutrition, plant-soil-microbe consortium, stress resilience

## Abstract

Arbuscular Mycorrhizal (AM) symbiosis is integral to sustainable agriculture and enhances plant resilience to abiotic and biotic stressors. Through their symbiotic association with plant roots, AM improves nutrient and water uptake, activates antioxidant defenses, and facilitates hormonal regulation, contributing to improved plant health and productivity. Plants release strigolactones, which trigger AM spore germination and hyphal branching, a process regulated by genes, such as *D27*, *CCD7*, *CCD8*, and *MAX1*. AM recognition by plants is mediated by receptor-like kinases (RLKs) and LysM domains, leading to the formation of arbuscules that optimize nutrient exchange. Hormonal regulation plays a pivotal role in this symbiosis; cytokinins enhance AM colonization, auxins support arbuscule formation, and brassinosteroids regulate root growth. Other hormones, such as salicylic acid, gibberellins, ethylene, jasmonic acid, and abscisic acid, also influence AM colonization and stress responses, further bolstering plant resilience. In addition to plant health, AM enhances soil health by improving microbial diversity, soil structure, nutrient cycling, and carbon sequestration. This symbiosis supports soil pH regulation and pathogen suppression, offering a sustainable alternative to chemical fertilizers and improving soil fertility. To maximize AM ’s potential of AM in agriculture, future research should focus on refining inoculation strategies, enhancing compatibility with different crops, and assessing the long-term ecological and economic benefits. Optimizing AM applications is critical for improving agricultural resilience, food security, and sustainable farming practices.

## ﻿Introduction

Today, agriculture faces significant challenges due to abiotic stresses, including drought, salinity, heavy metal toxicity, and extreme temperatures. These stressors disrupt essential plant physiological processes such as nutrient uptake, water balance, and metabolic functions, leading to severe reductions in crop productivity (Ahmed et al. 2021; Hayat et al. 2023). Current estimates suggest that abiotic stresses contribute to up to a 70% decline in agricultural yields globally, posing a substantial threat to food security, as the world’s population is projected to exceed 9.3 billion by 2050, necessitating a 60% increase in food production (Muhammad et al. 2016; Ashokan et al. 2024). Compounding these challenges, climate change has amplified the frequency and intensity of weather events, further reducing crop resilience (Jin et al. 2024a). Unsustainable farming practices, such as excessive fertilizer use and intensive tillage, exacerbate soil degradation, leading to reduced soil fertility, water retention, and structural stability (Kuila and Ghosh 2022; Abdullahi 2023).

Arbuscular mycorrhizal fungi (AM) are symbiotic fungi that form mutualistic relationships with the roots of most terrestrial plants, providing a natural and sustainable means of enhancing plant resilience and productivity under abiotic stress. AM extends hyphal networks into the soil, significantly increasing the effective surface area of roots for water and nutrient absorption. These networks facilitate the uptake of essential nutrients, such as phosphorus (P), nitrogen (N), potassium (K), and zinc (Zn), reducing the need for chemical fertilizers while supporting plant growth in nutrient-poor soils (Salvioli di Fossalunga and Novero 2019; Corratgé-Faillie et al. 2023; Ahmed et al. 2024b). In addition to nutrient acquisition, AM improves root hydraulic conductivity under drought stress, maintains osmotic balance under saline conditions, and detoxifies heavy metals in contaminated soils through chelation and sequestration (Calvo-Polanco et al. 2014; Gong et al. 2023). In addition to their physical contributions, AM also influences plant metabolism and stress responses at the molecular level. Symbiosis with AM triggers transcriptional reprogramming in plants by activating genes related to nutrient transport, hormonal signaling, and stress adaptation (Gao et al. 2023; Gong et al. 2023). These changes enable plants to cope better with abiotic stresses, highlighting the multifaceted benefits of AM in agricultural systems.

The establishment and function of AM symbiosis are governed by complex molecular signaling pathways that coordinate fungal colonization and nutrient exchange. The interaction begins with the release of strigolactones from plant roots, which stimulates fungal spore germination and hyphal branching (Maclean et al. 2017; Slimani et al. 2024). These signals are recognized through the common symbiosis (SYM) signaling pathway, which involves receptor-like kinases, such as LysM RLKs. This activation triggers calcium signaling cascades mediated by CCaMK and CYCLOPS proteins, which are essential for transcriptional reprogramming and the establishment of symbiosis (Maclean et al. 2017). Key regulatory genes play pivotal roles in this process. For instance, RAM1 acts as a master regulator of mycorrhizal development by controlling the genes responsible for arbuscule formation and nutrient transport (Pimprikar et al. 2016; Slimani et al. 2024). Phosphate uptake, a hallmark of AM symbiosis, is facilitated by the PHT1 family of transporters, which are specifically expressed during colonization (Rui et al. 2023). Dysfunction of these transporters impairs arbuscule formation, highlighting their critical role in nutrient exchange (Salvioli di Fossalunga and Novero 2019). AM contributes to nitrogen transfer through a series of metabolic steps that involve both direct uptake and conversion. Initially, AM absorbs nitrogen in the form of nitrate (NO_3_^−^) or ammonium (NH_4_^+^) from the soil via its extraradical mycelium (ERM). For nitrate, the external hyphae directly take up and convert it to ammonium through a reduction process catalyzed by nitrate reductase enzymes (Tian et al. 2010; Bücking and Kafle 2015). Nitrate is first reduced to nitrite (NO_2_^−^) and then further reduced to ammonium (NH_4_^+^). This conversion was more efficient in AM than in non-mycorrhizal fungi, reflecting their adaptation to nitrogen acquisition (Govindarajulu et al. 2005; Bücking and Kafle 2015). Once ammonium is absorbed, it is assimilated into amino acids, with arginine as the major nitrogen carrier. The enzymes carbamoyl-phosphate synthetase (CPS) and argininosuccinate lyase (AL) facilitate the synthesis of arginine in the ERM (Tian et al. 2010; Bücking and Kafle 2015). Arginine is then transported to the intraradical mycelium (IRM) where it can be broken down into free ammonium and other amino acids at the symbiotic interface. These nitrogen compounds are released for uptake by the plant through specific transporters, including ammonium transporters (AMTs) and nitrate transporters (NPFs), which are upregulated in response to AM colonization (Luo et al. 2023; Wang et al. 2023a). This tightly regulated process ensures that nitrogen is available to the plant in an efficient and controlled manner, supporting plant growth while maintaining symbiotic balance (Govindarajulu et al. 2005; Hodge and Fitter 2010).

Phytohormones play an essential role in regulating the development and maintenance of AM symbiosis, thereby influencing colonization, arbuscule formation, and stress adaptation. For example, auxins are critical in the early stages of colonization by regulating strigolactone synthesis, which in turn promotes fungal hyphal branching and enhances the establishment of a symbiotic relationship (Liao et al. 2018; Mitra et al. 2022). Strigolactones themselves act as key signaling molecules during the presymbiotic phase, modulating fungal metabolism and auxin flux through the regulation of auxin transporters (Liao et al. 2018). Additionally, cytokinins (CKs) are involved in AM interactions, and increased CK levels in the host plant often correlate with enhanced AM colonization. AM fungi may produce CKs, stimulate host CK biosynthesis, or inhibit CK degradation to maintain a positive feedback loop that promotes colonization (Goh et al. 2019). Conversely, gibberellic acid (GA) was modulated by AM colonization, with higher levels often observed in mycorrhizal roots. Although initially thought to inhibit AM symbiosis, GAs plays a complex role, with their effects varying depending on the presence of other hormones and environmental conditions (Liao et al. 2018). Jasmonic acid (JA) also plays a role in AM interactions, with its effects ranging from positive to inhibitory, depending on its concentration and environmental conditions (Foo et al. 2013). Under stress conditions, JA integrates hormonal signals such as gibberellic acid and cytokinins to modulate plant responses and optimize symbiosis (Foo et al. 2013; Jang et al. 2020). Salicylic acid (SA) has been shown to improve AM symbiosis, particularly under stress conditions such as salt stress, by enhancing plant growth and activating metabolic pathways to prioritize reproductive functions (Garg and Bharti 2018).

The interplay between these hormones becomes even more intricate under abiotic stresses such as drought and heavy metal toxicity. For instance, under drought stress, abscisic acid (ABA) levels increase, which in turn supports AM colonization and functionality (Tang et al. 2022). Similarly, under heavy metal stress, AM can induce IAA, ABA, and GA synthesis to enhance plant resistance (Wang et al. 2023a). Hormonal crosstalk is highly dynamic, as it involves protein stability, gene transcription, and hormonal homeostasis, with various pathways interacting at multiple levels (Aerts et al. 2021). At the molecular level, AM symbiosis enhances the expression of key genes involved in stress responses and detoxification, such as heavy metal ATPase (HMA) genes, which help alleviate Cu toxicity (Wang et al. 2023a) This complex hormonal regulation ensures that AM can maintain plant health under challenging environmental conditions, showcasing the dynamic relationship between hormones and AM that underpins successful symbiosis.

Despite significant progress in the understanding of AM -plant interactions, critical gaps remain in both fundamental knowledge and practical applications. For example, the molecular mechanisms underlying secondary metabolite production and transcriptional regulation during AM symbiosis have not been fully elucidated (Gao et al. 2023). Similarly, the interplay between phytohormones during stress-specific responses requires further investigation, particularly in the context of hormonal crosstalk (Jang et al. 2020; Mitra et al. 2022). On the practical side, environmental conditions such as high soil phosphate levels and widespread use of agrochemicals significantly reduce AM colonization rates, limiting their effectiveness in field applications (Salvioli di Fossalunga and Novero 2019; Kuila and Ghosh 2022; Ahmed et al. 2024c). Additionally, the long-term ecological effects of AM inoculation on soil health, microbial diversity, and carbon sequestration remain underexplored (Lutz et al. 2023). Addressing these gaps is critical for optimizing AM-based interventions for sustainable agriculture. This review aims to provide a comprehensive overview of the molecular and physiological mechanisms underlying AM -plant interactions, with a focus on their role in enhancing plant resilience to abiotic stress. Specifically, it explores the regulatory pathways and hormonal crosstalk that govern AM symbiosis, examines the practical challenges of AM inoculation in agricultural systems, and identifies the critical research gaps. By synthesizing the current knowledge and proposing future directions, this review underscores the transformative potential of AM in advancing sustainable agriculture and ensuring global food security.

## ﻿Cellular and molecular dynamics of AM symbiosis with plants

**Figure 1.**
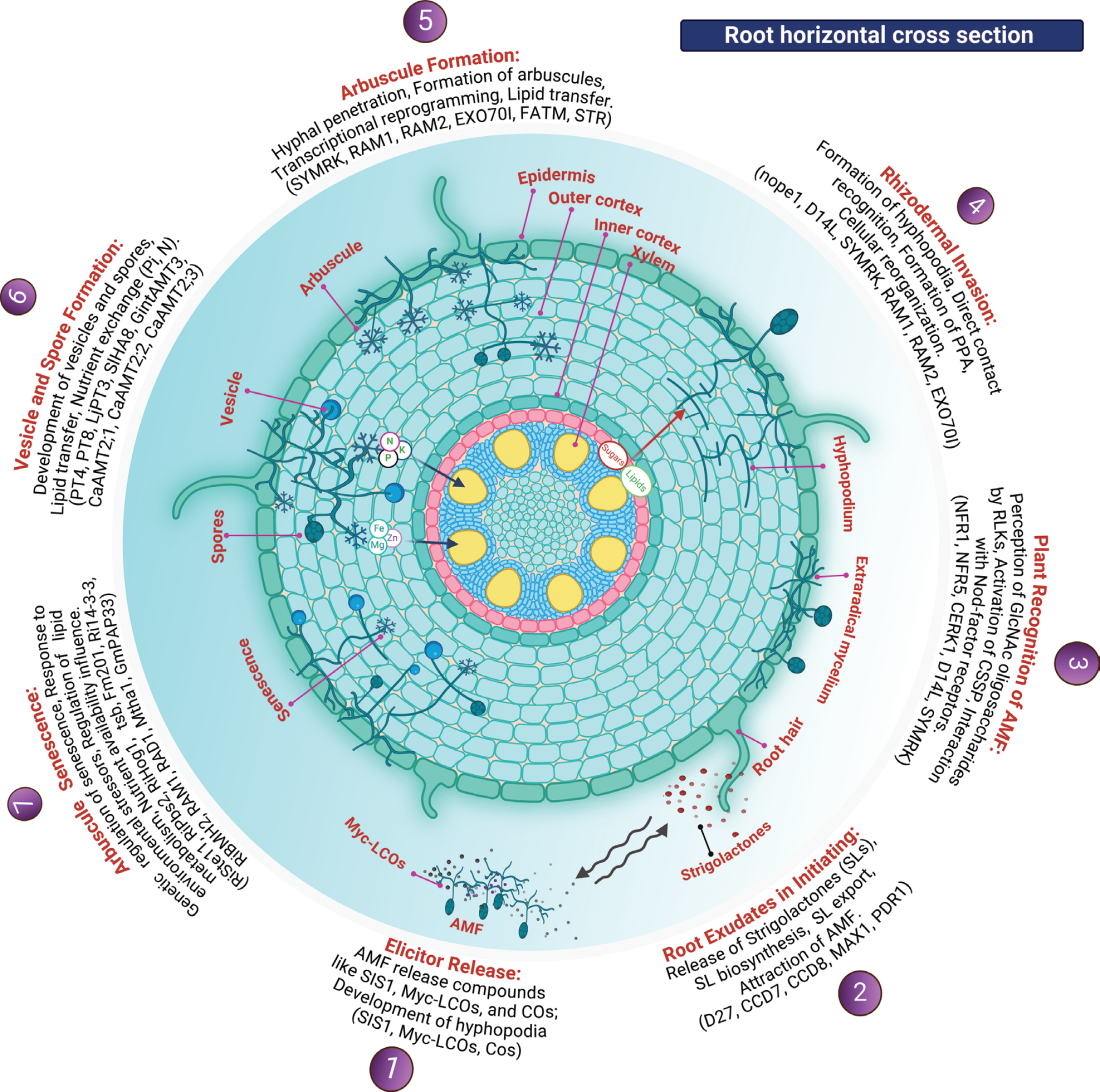
The sequential stages of symbiosis between plants and AM , starting from signal exchange with the release of strigolactones by the plant, to hyphal branching and colonization, leading to the establishment of arbuscules within root cortical cells for nutrient exchange.

The symbiotic relationship between AM and plants is a highly sophisticated biological interaction involving a sequence of molecular and cellular events that leads to the formation of functional symbiotic structures, nutrient exchange, and long-term maintenance of the relationship. Fig. 1 provides a comprehensive overview of the stages involved in AM colonization, from the initial contact with plant roots to the formation of arbuscules, followed by nutrient exchange and eventual senescence of the symbiotic structures.

### ﻿Root exudates and elicitor release in AM symbiosis

AM symbiosis is initiated when the host plant secretes root exudates that signal and attracts AM hyphae (Fig. 1). These exudates, including strigolactones (SLs) such as 5-deoxystrigol and orobanchol, stimulate fungal metabolism and promote hyphal branching, thereby increasing the likelihood of AM encountering plants (Mitra et al. 2021; Moreno et al. 2021). SL export into the rhizosphere is facilitated by the ABC transporter PDR1, which creates a concentration gradient that guides AM (Rehman et al. 2021). Under phosphorus deficiency, genes involved in SL biosynthesis, such as *D27*, *PDR1*, and *MAX1*, were upregulated to attract fungi (Waters et al. 2017; Yang Y. et al. 2023). SL production varies across plant species. For example, sunflower (*Helianthusannuus*) and black oats (*Avenastrigosa*) accumulate carlactones, which also promote AM branching (Vangelisti et al. 2018). In addition to SLs, plants release phenolic acids and flavonoids (e.g., chrysin, quercetin, and rutin), which act as presymbiotic signals. These compounds stimulate AM spore germination and root colonization by inducing the synthesis of Myc-lipochitooligosaccharides (Myc-LCOs) (Lidoy et al. 2023). Hormones, such as auxins, ABA, methyl jasmonate, and brassinosteroids (BRs), regulate root hair growth and development, which in turn enhances AM colonization (Gutjahr 2014; Liu et al. 2018). Mycorrhizal inoculation increased root hair density, length, and diameter through hormone modulation. Under drought stress, AM inoculation elevates root concentrations of ABA, IAA, and BRs, thereby promoting drought adaptation (Zhang et al. 2019a). Additionally, proteins such as PIN1 and LAX3, which regulate auxin transport, further facilitate root colonization and symbiosis (Liu et al. 2018; Wang et al. 2021b).

Upon reaching the root surface, AM hyphae secrete Myc-LCOs, which are crucial for the initiation of symbiosis (Fig. 1). These molecules are recognized by LysM receptor-like kinases (RLKs), such as CERK1 and LYK8, which play distinct roles in symbiotic signaling. In *Medicagotruncatula*, the CERK1/LYK8 complex is necessary for mycorrhizal colonization, activating downstream transcription factors, such as NIN-like proteins (NLPs) and RAM1, to regulate symbiosis gene expression and facilitate successful mycorrhizal establishment(Guillotin et al. 2016; Zhang et al. 2024a). The recognition of short-chain chitin oligomers (COs) secreted by AM triggers calcium (Ca²^+^) spiking, which activates the symbiotic signaling cascade, including genes in the common SYM pathway (*DMI1/DMI2*), which is essential for fungal root colonization(Genre et al. 2013; Zhang et al. 2024a). Additionally, *RAM1* induces genes, such as *STR*, *STR2*, and *PT4*, which are critical for arbuscule development and nutrient exchange (Rich et al. 2017).

AM and rhizobia produce structurally similar lipochitooligosaccharides (LCOs), which are recognized by LysM receptor-like kinases in plant roots. Both Myc-LCOs and Nod-LCOs contain N-acetyl glucosamine and fatty acid modifications, but their host specificity is determined by their unique decorations. CERK1/LYK proteins mediate the perception of both types of LCOs. Despite sharing a similar recognition mechanism, their downstream signaling pathways are regulated differently to ensure distinct symbiotic outcomes (Lace and Ott 2018; Wang et al. 2022a). Additionally, Myc-LCOs trigger calcium spiking, which activates MtSymRK (Symbiosis Receptor Kinase), a key player in the symbiotic signaling cascade (Genre et al. 2013). Furthermore, AM secretes the protein SIS1 (SL-induced putative secreted protein 1), which is essential for root colonization. Downregulation of *SIS1* results in reduced infection and smaller arbuscules (Lanfranco et al. 2018). AM also releases short-chain chitooligosaccharides (COs), which together with Myc-LCOs, facilitate early symbiosis stages(Ho-Plágaro and García-Garrido 2022). These oligosaccharides are structurally similar to GlcNAc-based molecules produced by rhizobia and pathogenic fungi, raising questions regarding signal specificity. The composition and proportion of GlcNAc-based signals in AM germinating spore exudates may help distinguish AM from other microorganisms (Buendia et al. 2018; Lace and Ott 2018). Overall, root exudates (SLs), flavonoids, hormones, and AM-secreted compounds such as SIS1 and oligosaccharides create a complex network of chemical signals that regulate AM growth, root colonization, and the initiation of symbiosis.

### ﻿AM root colonization: invasion and hyphopodia formation

Following recognition by AM, fungal hyphae form hyphopodia to anchor to the root epidermis (Fig. 1), a process regulated by plant Rho GTPases and cytoskeletal rearrangements (Kiirika et al. 2012). Notably, AM can form hyphopodia in SL biosynthetic mutants, suggesting that this process can be triggered by direct contact recognition independent of SL signaling (Mashiguchi et al. 2023). Additionally, hyphopodium formation in *Verticilliumdahliae* is regulated by a MAP kinase cascade (VdSte11-VdSte7-VdKss1 pathway), cAMP-mediated signaling, and VdNoxB/VdPls1-dependent ROS-Ca^2+^ pathway (Zhao et al. 2016; Sun et al. 2019; Ye et al. 2023). Studies on maize and rice mutants, including those lacking *nope1* and *D14L*, further suggesting that presymbiotic communication is essential for hyphopodia formation, and (Mitra et al. 2021; Mashiguchi et al. 2023) lipid signaling, involving lipid transfer proteins (LTPs) like *LTP1*, also facilitates AM–plant interactions (Amador et al. 2021). Once hyphopodia is established, AM hyphae penetrate the root epidermis through the pre-penetration apparatus (PPA), a tube-like structure that aids fungal entry (Fig. 1). This process is regulated by MLO proteins, with *VpMLO13* in *Vitispseudoreticulata*, which plays a key role in modulating plant defense responses, similar to the role of *MLO1* in *Arabidopsis* (Huang et al. 2023). The formation of PPAs resembles infection thread formation during rhizobial colonization in *Pipernigrum*, suggesting common signaling pathways for both processes (Trindade et al. 2019). SYMRK, a receptor kinase, mediates interactions with both AM and rhizobia, triggering epidermal calcium spiking, which is critical for intracellular accommodation and root development (Krönauer and Radutoiu 2021; Roy Choudhury and Pandey 2022). Although the role of *D14L* in AM perception remains unclear, studies on CSSP mutants in rice suggest that AM colonization can still occur, although fungal structures are not fully developed in *D14L* mutant roots (Chiu et al. 2018). These findings suggest that hyphopodia formation and AM root colonization are complex and involve a network of signaling molecules, receptors, and proteins that coordinate the symbiotic relationship.

### ﻿Arbuscule formation

Arbuscule formation begins with AM hyphae penetrating root cortical cells non-destructively, guided by PPA (Fig. 1), leading to the formation of highly branched arbuscules for efficient nutrient transfer (Genre et al. 2008). Key genes involved in arbuscule development include *SYMRK*, which facilitates AM colonization and arbuscule formation (Tan et al. 2013), and *RAM1*, a transcription factor that regulates plant defense suppression and root cortical cell reprogramming (Irving et al. 2022). Overexpression of *RAM1*, shown to increase arbuscule density and AM-inducible gene expression in dicots and monocots, underscores its pivotal role in mycorrhization (Müller et al. 2020). *EXO70I*, regulated by *RAM1*, is also critical for arbuscular branching and development (Park et al. 2015). In the absence of *RAM1*, as in the ram1-3 mutant, arbuscule branching is impaired, highlighting the importance of *EXO70I* in arbuscule formation (Park et al. 2015). Another key gene, *RAM2*, encodes a glycerol-3-phosphate acyltransferase essential for lipid biosynthesis, particularly the cutin monomers required for arbuscule formation (Bravo et al. 2017). Mutants of *OsRAM2* in rice show defective arbuscules and low colonization rates, further implicating *RAM2* in lipid transfer and signaling at the root surface (Dai et al. 2022; Liu et al. 2022). The secretion of 2-monoacylglycerols (2MGs), driven by *RAM2* and associated with other proteins such as *FATM* and *STR*, emphasizes the importance of lipid signaling in arbuscule functions (Müller et al. 2020; Kameoka and Gutjahr 2022). Together, the coordinated actions of these genes—*SYMRK*, *RAM1*, *KIN3*, *EXO70I*, *RAM2*, *OsRAM2*, *FATM*, and *STR*—govern the complex molecular mechanisms that drive successful arbuscule formation and AM -plant symbiosis, critical for nutrient exchange and plant health.

### ﻿Arbuscule senescence

Arbuscules are the primary sites for nutrient exchange in AM symbiosis, and their senescence is regulated by a complex interplay of genetic, physiological, and environmental factors (Fig. 1). The HOG1-MAPK cascade genes (*RiSte11*, *RiPbs2*, *RiHog1*) play crucial roles in arbuscule development and senescence under stress conditions. Silencing of these genes impairs arbuscule formation, particularly under drought stress (Wang et al. 2023b). Similarly, the tomato *tsb* gene, encoding a microtubule-associated protein, is involved in arbuscule turnover and senescence, as its overexpression leads to fully developed but inactive arbuscules (Ho-Plágaro et al. 2021). 14-3-3-like proteins (e.g., *Fm201*, *Ri14-3-3*, *RiBMH2*) and GRAS-type transcription factors (e.g., *RAM1*, *RAD1*) are also critical for maintaining arbuscule functionality, with their mutations leading to degenerated arbuscules (Xue et al. 2015; Sun et al. 2018). Moreover, *Mtha1* gene, which encodes an H(+)-ATPase, helps prevent premature senescence by maintaining arbuscular integrity (Franken et al. 2007). Environmental stressors such as low pH and drought can accelerate arbuscular senescence. Low pH inhibits arbuscule formation and lipid transfer from the host to the fungus, thereby promoting arbuscule degeneration (Roth et al. 2019; Feng et al. 2020). Drought stress induces oxidative damage in nodules; however, this effect is mitigated through AM symbiosis, suggesting that oxidative stress is a key factor in premature senescence (Serova and Tsyganov 2014). Regulation of lipid metabolism, particularly through purple acid phosphatase (*GmPAP33*), plays a crucial role in phospholipid hydrolysis during arbuscule senescence (Li et al. 2019). Additionally, nutrient starvation and oxidative stress exacerbate arbuscule degradation (Daher et al. 2017). Nutrient availability significantly influences arbuscule longevity. While high phosphorus levels inhibit new arbuscule formation and cause abnormal hyphal branching, preexisting arbuscules remain functional, whereas low phosphorus availability enhances arbuscule formation and nutrient exchange, promoting AM symbiosis (Schildhauer et al. 2008; Carbonnel and Gutjahr 2014; Roth et al. 2019). Nitrogen availability further affects arbuscule maintenance; the addition of nitrogen can delay leaf senescence and support arbuscule longevity by ensuring a favorable nutrient environment (Schildhauer et al. 2008; Carbonnel and Gutjahr 2014). However, nutrient imbalances, such as high N and low P, can accelerate senescence owing to insufficient P uptake (Schildhauer et al. 2008; Carbonnel and Gutjahr 2014).

### ﻿Vesicle and spore formation and nutrient transport in AM symbiosis

In AM symbiosis, the formation of vesicles and spores is crucial for nutrient exchange between plants and fungi (Fig. 1). Plants provide glucose and lipids to AM, while AM supplies inorganic nutrients to plants, highlighting the mutual dependency of both partners. AM, particularly those from the phylum *Glomeromycota* (except *Gigasporaceae*), form lipid storage vesicles, which indicate lipid transfer from the host. This process is further supported by reduced vesicle formation in plants with mutations in the genes involved in lipid metabolism (Mathu Malar et al. 2022). In return, AM produces asexual spores rich in storage lipids, such as triacylglycerol (TAG), which are essential for spore germination and early growth when detached from the host (Keymer et al. 2017). As AM cannot synthesize long-chain fatty acids de novo, they rely on the host for lipid supply, with genes such as *KAS III* and *RAM2* playing crucial roles in lipid transfer (Keymer et al. 2017; Lanfranco and Bonfante 2023). AM also transports phosphorus, nitrogen, and other essential elements from the soil to plants via specialized transporters located in the periarbuscular membrane (PAM) (Chiu and Paszkowski 2019). Phosphate transporters such as *PT4* and *PT8* are vital for phosphorus uptake at the symbiotic interface and serve as markers for cells with functional arbuscules (Cosme et al. 2016; Cope et al. 2022; Perotto and Balestrini 2024). Additionally, the H^+^ gradient generated by *SlHA8*, a PAM-localized H^+^-ATPase in tomatoes, is crucial for nutrient uptake (Liu et al. 2020). Ammonium transporters (AMTs) play significant roles in AM symbiosis. In *Rhizophagusirregularis*, *GintAMT3* is expressed in the intraradical mycelium and functions as a low-affinity transporter of ammonium at the arbuscular site (Calabrese 2016). In *Brassicanapus*, *AMT3;1* is the primary transporter for mycorrhizal ammonium transfer (Dai et al. 2023), whereas in *Capsicumannuum*, genes such as *CaAMT2;1* are upregulated during AM colonization, indicating their involvement in ammonium uptake (Fang et al. 2023). Similarly, in *Triticumaestivum*, the expression of *TaAMT1.2* is downregulated by AM colonization in response to nitrogen signals from AM hyphae (Tian et al. 2017). Overall, the formation of vesicles and spores by AM, along with the intricate nutrient transport mechanisms, underscores the complexity of AM symbiosis, in which lipid and nutrient exchange are essential for the growth and survival of both partners. Understanding the interplay between genetic, physiological, and environmental factors that regulate arbuscule senescence is crucial for optimizing AM symbiosis. This knowledge can enhance plant nutrient uptake and stress resilience, both of which are vital for improving agricultural sustainability.

## ﻿Hormonal regulation of AM symbiosis through integrative signaling mechanisms and stress adaptation

**Figure 2.**
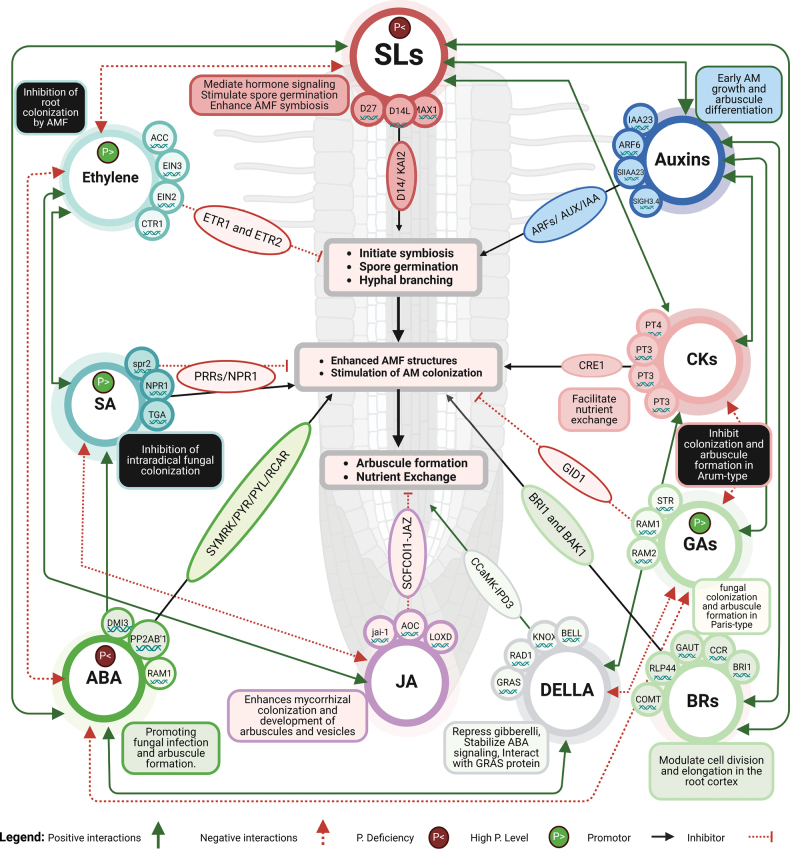
The intricate hormonal signaling pathways and interactions of hormones and key genes involved in AM symbiosis.

AM symbiosis plays a vital role in the enhancement of plant nutrient uptake and stress resilience. Recent studies have underscored the complex interplay between various plant hormones and the signaling pathways that regulate AM symbiosis, from initial recognition and signaling to arbuscule formation and eventual senescence (Fig. 2). Hormones such as strigolactones, cytokinins, auxins, gibberellins, ethylene, jasmonic acid, and abscisic acid orchestrate different stages of the symbiotic process, influencing key events such as fungal recognition, hyphal branching, root development, and arbuscule maturation. Understanding these molecular mechanisms is critical for improving the efficiency of AM symbiosis and offers insights into enhancing agricultural sustainability and stress adaptation in plants.

### ﻿Strigolactones (SLs): Dual roles in symbiosis and root architecture

Strigolactones (SLs) are carotenoid-derived phytohormones essential for the initiation of symbiosis with AM (Fig. 2). The biosynthesis of SLs involves key genes, such as *DWARF27* (*D27*) and *MORE AXILLARY GROWTH 1* (*MAX1*). D27 catalyzes the isomerization of all-trans-β-carotene to 9-cis-β-carotene, initiating the SL biosynthetic pathway, whereas *MAX1* is involved in the later stages of SL biosynthesis, where it converts carotenoid-derived intermediates into bioactive SLs (Waters et al. 2017; Yang et al. 2023). These active SLs play a crucial role in signaling the presence to a host plant, thereby facilitating fungal spore germination and establishing a symbiotic relationship (Liao et al. 2018; Kee et al. 2023). This interaction is mediated by receptors such as D14, which specifically perceive SLs, and KAI2, which respond to smoke-derived compounds called karrikins (Kodama et al. 2023). The receptors D14 and KAI2, in coordination with the F-box protein MAX2, mediate signal transduction by triggering proteasomal degradation of SMAX1 and SMXL2 proteins (Khosla et al. 2020; Tal et al. 2023). This degradation promotes hyphal growth and enhances fungal colonization, thereby facilitating the establishment of arbuscular mycorrhizal symbiosis. The presence of SLs is particularly pronounced under nutrient-deficient conditions, such as low phosphate levels, which enhances their role in facilitating mycorrhization (Kodama et al. 2022). Additionally, the auto-regulation of mycorrhization involves transcription factors, such as *NSP1* and *NSP2*, which control SL synthesis and fungal colonization, ensuring a balanced symbiotic interaction (Guillotin 2016). Moreover, SLs are involved in a complex network of phytohormonal interactions, including auxin, cytokinin, and ethylene, which modulate root growth and optimize the conditions for symbiotic relationships (Shafi et al. 2022; Sun et al. 2022). The exudation of strigolactones (SLs) not only attracts AM but also supports fungal development and root colonization, thereby enhancing plant nutrient uptake, particularly phosphorus and nitrogen.

### ﻿Cytokinins (CKs): Coordinating root development and mycorrhizal symbiosis

Cytokinins (CKs) are crucial phytohormones that are involved in the regulation of plant growth, development, and symbiotic interactions, including those with AM. In AM symbiosis, CKs stimulate AM colonization, with increased levels of active CKs correlating with enhanced AM structures in the roots (Goh et al. 2019). Conversely, reduced CK levels led to diminished colonization, indicating their stimulatory role in symbiosis. In transgenic tobacco with altered CK levels, shoot CKs positively influence AM fungal development and the expression of AM-responsive genes, such as *NtPT4*, which is involved in phosphate transport, highlighting the role of CKs in nutrient exchange between the plant and the fungi (Cosme et al. 2016). CK transporters, such as *LjPup1* and *LjPT3* in *Lotusjaponicus*, export CKs from nodules to shoots, contributing to shoot growth and overall plant development (Maeda et al. 2006; Chen et al. 2022b). The CK receptor homolog MtCRE1 in *Medicagotruncatula* regulates lateral root formation and nodulation, and its loss leads to increased lateral roots and reduced nodulation (Gonzalez-Rizzo et al. 2006). It also plays a critical role in symbiosis with *Sinorhizobiummeliloti* by regulating early CK response genes such as *MtRR1* and *MtRR4* (Gonzalez-Rizzo et al. 2006). Although *MtCRE1* is involved in the crosstalk between plant CKs and bacterial Nod factors, it is not part of the common symbiotic pathway shared by rhizobial and AM symbiosis. Furthermore, in response to *Aphanomyceseuteiches* infection, *cre1* mutants showed enhanced lateral root formation and resistance, highlighting the role of CK signaling in stress response (Laffont et al. 2015). Building on the importance of cytokinin (CK) signaling in symbiosis, the ATP-binding cassette (ABC) transporter *MtABCG56* plays a crucial role in the early stages of nodulation. Expressed in both the roots and nodules, *MtABCG56* exports bioactive CKs in an ATP-dependent manner, facilitating CK distribution across the plasma membrane. Disruption of this transporter impaired arbuscule formation, further underscoring the significance of CK transport in both AM and rhizobial symbiosis (Jarzyniak et al. 2021). These findings highlight the importance of CKs in coordinating plant growth, symbiotic interactions, and disease resistance, with CK transporters and receptors playing central roles in mediating these processes.

### ﻿Auxins (AUX): Shaping root morphology for Mycorrhizal colonization

Auxin plays a pivotal role in the establishment and maintenance of AM symbiosis by modulating various stages of plant-AM interactions, particularly through the regulation of arbuscule formation and mycorrhization (Fig. 2). Indole-3-acetic acid (IAA), a key auxin compound, accumulates in plant roots upon AM colonization, promoting the differentiation of arbuscules and enhancing mycorrhization at optimal concentrations (Chen et al. 2022a; Li et al. 2023a). This hormone is essential for early fungal growth, with disruptions in auxin signaling leading to inhibited AM colonization and impaired root development (Ludwig-Müller and Güther 2007). Several key genes, including *AUXIN RESPONSE FACTORS* (ARFs) and *AUXIN/INDOLE-ACETIC ACIDS* (*AUX/IAAs*), play crucial roles in regulating auxin function in AM symbiosis (Jentschel et al. 2007; Yurkov et al. 2017). For instance, *SlARF6* negatively regulates AM colonization, whereas *SlIAA23* promotes colonization by interacting with *SlARF6*, thereby enhancing phosphorus uptake and modulating SL synthesis (Li et al. 2023a). Additionally, the *GH3* gene family, especially *SlGH3.4*, is involved in maintaining auxin homeostasis. Loss of *SlGH3.4* leads to increased free IAA levels and higher arbuscule incidence, further promoting mycorrhization (Chen et al. 2022a). Auxin accumulation in specific cells is crucial for the localized development of symbiotic structures, facilitated by both plant and fungal auxin transporters and biosynthetic activities (Barker and Tagu 2000). The regulation of various auxin compounds, such as IAA, indole-3-butyric acid (IBA), and phenylacetic acid (PAA), throughout the different stages of AM development adds complexity to the role of auxin in the symbiotic process. These auxin derivatives contribute to the fine-tuned regulation of fungal growth, root colonization, and establishment of a symbiotic relationship (Liao et al. 2018). Overall, the coordinated regulation of auxin and its derivatives plays a fundamental role in the successful establishment of AM symbiosis, thereby enhancing plant growth, nutrient uptake, and stress resilience.

### ﻿Brassinosteroids (BRs): Integrating growth and symbiotic functions

Brassinosteroids (BRs) are crucial plant steroid hormones that regulate a wide range of growth and developmental processes, including the formation of arbuscules in mycorrhizal symbiosis (Fig. 2). By controlling cell division and elongation, brassinosteroids (BRs) orchestrate gene regulatory networks, particularly in the root cortex, driving the transition from cell proliferation to elongation. This process, promoted by CH4-induced BR accumulation, enhances the expression of cell wall-related genes, including enzymes such as xyloglucan endotransglucosylase/hydrolase (XTH) and peroxidase, while reducing the cellulose, hemicellulose, and lignin content, thereby facilitating adventitious root formation (McGuiness et al. 2020; Li et al. 2023b). This shift also supports the accommodation of fungal hyphae within root cortical cells, which is a key step in establishing a successful symbiotic relationship with AM. The BR receptor *BRASSINOSTEROID INSENSITIVE1* (*BRI1*), which undergoes post-translational modifications, such as SUMOylation, plays a central role in regulating BR signaling and mediating plant responses to environmental stimuli (Naranjo-Arcos et al. 2023). *BRI1* interacts with *BAK1* (*BRI1-ASSOCIATED RECEPTOR KINASE 1*), a co-receptor that enhances BR signaling and promotes root development and arbuscule formation during AM symbiosis. This interaction is crucial for amplifying the BR signaling pathway, ensuring optimal plant growth and adaptation under varying environmental conditions (Li and Chory 1997; Chinchilla et al. 2009; Naranjo-Arcos et al. 2023). BRs also modulate cell wall properties by altering the expression of genes, such as *UAM*, *GAUT*, *COMT*, and *CCR*, which encode enzymes involved in cell wall loosening and restructuring (XTH and peroxidase). These enzymes facilitate cell expansion and elongation, further promoting root and arbuscular development (Rao et al. 2017; Li et al. 2023b). Proteins such as RLP44 integrate BR signaling with cell wall regulation to maintain homeostasis and control vascular cell fate, thus influencing the overall architecture of the root system (Garnelo Gómez 2018). Moreover, BRs interact with other plant growth regulators (PGRs), such as Aux, NO, and SLs, collectively promoting lateral root formation and increasing root surface area, which enhances arbuscule development and mycorrhizal colonization (Siddiqi and Husen 2021; Altamura et al. 2023; Nolan et al. 2023). Hormonal crosstalk regulates gene expression, ensuring efficient nutrient exchange, colonization, and stress resilience, which are critical for plant health and growth under diverse environmental conditions.

### ﻿Salicylic Acid (SA): Influence on AM symbiosis through stress regulation

Salicylic acid (SA) plays a complex and context-dependent role in AM symbiosis, influencing both the establishment and functionality of this relationship (Fig. 2). Under salt stress, SA enhances AM root colonization, promoting arbuscule and vesicle formation, suggesting a supportive role in adverse conditions (Garg and Bharti 2018). However, elevated SA levels can delay AM colonization, as observed in *spr2* mutant tomato plants, owing to the upregulation of SA-related defense genes (Casarrubias-Castillo et al. 2020). SA interacts antagonistically with jasmonic acid (JA) signaling, and elevated SA levels can suppress JA responses that are critical for AM symbiosis (Hickman et al. 2019). The key genes involved include *NONEXPRESSOR OF PATHOGENESIS-RELATED GENES 1* (*NPR1*), which regulates SA-mediated defenses and modulates JA-dependent pathways (Abd El Rahman et al. 2012). Elevated SA levels upregulate pathogenesis-related (*NPR*) genes, including chitinases and β-1,3-glucanases, which degrade fungal cell walls and inhibit fungal colonization (dos Santos and Franco 2023). Additionally, *NPR1* interacts with TGA transcription factors to activate SA-responsive genes while suppressing the JA-responsive genes critical for AM signaling (Abd El Rahman et al. 2012). SA modulates plant responses to phosphate availability. Higher levels of phosphate (Pi) in mycorrhizal roots repress symbiotic gene expression and AM colonization, affecting key genes involved in carotenoid and SLs biosynthesis, as well as phosphate transporters, through the activation of defense mechanisms such as chitinases (Breuillin et al. 2010). Furthermore, SA can inhibit pathogen growth at higher concentrations, indirectly affecting AM by altering the plant defense status (Herrera Medina et al. 2003). The role of SA in AM symbiosis is also modulated by its interaction with other hormones. In ABA-deficient tomato mutants, the application of exogenous ABA restores both fungal activity and arbuscule functionality, indicating a crosstalk between SA, ABA, and ethylene signaling pathways in regulating AM symbiosis (Herrera-Medina et al. 2007). Moreover, SA directs carbohydrate metabolism towards reproductive functions, influencing nutrient exchange between plants and fungi (Garg and Bharti 2018). Finally, SA likely affects cytoskeletal remodeling in root cells, influencing arbuscule development and turnover by modulating microtubule-associated proteins (Herrera Medina et al. 2003; Ho-Plágaro et al. 2021). Thus, while SA can enhance AM symbiosis, its role is context-dependent, balancing the promotion and inhibition of arbuscule formation through various biochemical and physiological pathways.

### ﻿Gibberellins (GAs): Regulation of fungal colonization and arbuscule formation

Gibberellins (GAs) play a complex, context-dependent role in AM symbiosis, with varying effects on the AM type and plant species. In Arum-type AM symbiosis, GAs typically inhibits fungal colonization and arbuscule formation (Fig. 2). They suppress AM entry into the roots and downregulate key genes involved in symbiosis, such as *RAM1* and *RAM2* homologs (Tominaga et al. 2020b, 2020a). Conversely, in Paris-type AM symbiosis, seen in species such as *Eustomagrandiflorum*, is promoted by GAs, which enhance hyphopodium formation and fungal entry into roots (McGuiness et al. 2019; Tominaga et al. 2020b). This demonstrates that GAs plays species-specific roles in AM symbiosis. Infection by *Rhizophagusirregularis* leads to increased shoot growth and the expression of symbiosis-related genes, such as *RAM1* and *STR*. However, GA treatment produces differential effects across species: it decreases the expression of these genes in Arum-type *Lotusjaponicus* and Intermediate-type *Daucuscarota*, while increasing their expression in Paris-type *E.grandiflorum* (Tominaga et al. 2021). This variability emphasized the nuanced role of GAs in the regulation of AM symbiosis-related genes. Additionally, GAs interacts with other phytohormones, particularly ABA. In tomato, ABA antagonizes GAs by reducing bioactive GA levels via DELLA proteins, which suppresses GA signaling and promotes arbuscule formation (Martín-Rodríguez et al. 2016). DELLA proteins are repressed in response to GA signaling and are essential for arbuscule development, as shown by the *Mtdella1/Mtdella2* double mutant in *Medicagotruncatula*, in which impaired arbuscule formation is mimicked by exogenous GA (Floss et al. 2016). DELLA proteins also interact with *REQUIRED FOR ARBUSCULE DEVELOPMENT 1* (*RAD1*), further linking GA signaling to gene regulation during AM symbiosis (Floss et al. 2016; Liao et al. 2018). They regulate cytokinin metabolism by modulating the expression of *KNOX* and *BELL* transcription factors, which are crucial for nodule organogenesis and arbuscular development (Dolgikh et al. 2019). The impact of GAs on AM symbiosis is also influenced by Pi levels. High Pi concentrations can suppress AM symbiosis by increasing GA levels in mycorrhizal roots, suggesting that Pi regulates AM development through GA signaling (Nouri et al. 2021). Moreover, fluctuating GA levels can positively or negatively affect the expression of AM-related genes, thereby influencing fungal colonization in a spatially and temporally regulated manner (Bucher et al. 2014; Martín-Rodríguez et al. 2015). Thus, GAs performs diverse roles in AM symbiosis, with varying effects based on plant species, AM type, and hormonal interactions. The regulation of symbiotic genes and interactions with ABA and Pi highlights the complexity of GA’s role of GA in arbuscule formation.

### ﻿Ethylene: A key negative regulator of AM symbiosis

Ethylene plays a complex and primarily inhibitory role in the regulation of AM symbiosis (Fig. 2). Its signaling pathway is initiated when ethylene binds to a family of two-component histidine kinase receptors, such as ETR1 and ETR2. This binding inactivates these receptors, leading to inhibition of the negative regulator CTR1 (Martín-Rodríguez et al. 2011; Mukherjee and Ané 2011). This inactivation cascade allows the activation of *EIN2*, which stabilizes *EIN3*, the key transcription factor that mediates ethylene responses by regulating gene expression (Chen et al. 2005; Frankowski et al. 2008; Vidhyasekaran 2015). Studies have shown that in both monocots and eudicots, germinating spore exudates from AM induces the expression of symbiotic genes and promote root development, which are suppressed by ethylene signaling (Mukherjee and Ané 2011). This inhibitory role was further supported by research on tomato mutants with altered ethylene responses. Ethylene-insensitive mutants showed reduced inhibition of mycorrhization under high-phosphate conditions, whereas ethylene-overproducing mutants exhibited decreased mycorrhizal colonization (de Los Santos et al. 2011). Ethylene interacts with multiple signaling pathways to influence AM symbiosis, acting downstream of NO and hydrogen peroxide (H_2_O_2_) and upstream of JA and SA, which regulate secondary metabolite biosynthesis and may indirectly affect symbiosis (Ngom et al. 2020). Its effect is further modulated by ABA, as ABA-deficient plants show elevated ethylene levels, leading to reduced mycorrhization (Herrera-Medina et al. 2007; de Los Santos et al. 2011). This suggests that ABA, by regulating ethylene levels, plays a role in fine-tuning the balance of the inhibitory effects of ethylene on AM symbiosis. Thus, ABA fine-tunes the inhibitory effect of ethylene on AM colonization. Overall, ethylene primarily acts as a negative regulator of AM symbiosis by inhibiting root colonization, and its effect is shaped by interactions with ABA, NO, H_2_O_2_, JA, and SA.

### ﻿Jasmonic Acid (JA): Modulating mycorrhizal interactions and plant defense

Jasmonic acid (JA) signaling is crucial for modulating the establishment and function of AM symbiosis (Fig. 2). Among tomato plants, the JA-insensitive mutant *jai-1* showed increased susceptibility to fungal infection and accelerated colonization, highlighting JA’s role of JA in regulating the intensity and frequency of fungal colonization (Herrera-Medina et al. 2008). Systemic application of methyl jasmonate (MeJA), a JA precursor, enhances the expression of JA synthesis genes, such as *allene oxide cyclase* (*AOC*) and *lipoxygenase D* (LOXD), and boosts the activity of defense-related enzymes, including superoxide dismutase, guaiacol peroxidase, and catalase (Kamakshi et al. 2023). Furthermore, MeJA treatment leads to the accumulation of metabolites such as total phenolics, flavonoids, chlorogenic acid, caffeic acid, and lignin in both root and shoot tissues, which reduces mycorrhization by triggering mycorrhizal-induced resistance (Li et al. 2022; Kamakshi et al. 2023). In addition, MeJA affects fungal phosphate metabolism and arbuscule formation, further highlighting its role in regulating these processes (Herrera-Medina et al. 2008; Fattorini et al. 2018). However, in *Nicotianaattenuata*, ethylene was found to play a more significant role in AM interactions than JA, suggesting that the impact of JA on AM symbiosis may vary across plant species (Riedel et al. 2008). In contrast, garlic plants showed a synergistic effect of JA and AM inoculation, with JA enhancing mycorrhizal colonization and the development of arbuscules and vesicles (Regvar et al. 1996; Wasternack 2014). This variability across species underscores the complexity of JA’s role of JA in AM symbiosis. JA is also involved in the induction of N-fixing root nodules, and increases in response to mechanical disturbances, further linking it to symbiotic interactions (Hause et al. 2007). At the molecular level, JA signaling is mediated through the SCFCOI1-JAZ-co-receptor complex, which regulates gene expression by degrading JAZ proteins. This degradation allows transcription factors to activate JA-responsive genes (Wasternack 2014; Singh et al. 2015). This signaling network is critical not only for plant stress responses, but also for developmental processes, including AM symbiosis.

### ﻿Abscisic Acid (ABA): Enhancing symbiotic efficiency and stress tolerance

Abscisic acid (ABA) plays a multifaceted role in the regulation of AM symbiosis by influencing plant growth, stress responses, and symbiotic efficiency. At low concentrations, ABA enhanced AM colonization by promoting fungal infection and arbuscule formation, as observed in *Medicagotruncatula* and *Solanumlycopersicum* (Charpentier et al. 2014; Stec et al. 2016; Lou et al. 2021). This positive effect is mediated by the Protein Phosphatase 2A (PP2A) holoenzyme subunit PP2AB1, which is crucial for ABA’s role of ABA in AM colonization (Charpentier et al. 2014). ABA signaling begins with the binding of ABA to pyrabactin resistance (PYR) proteins (also known as PYL or RCAR), which inhibit type 2C protein phosphatases (PP2Cs), such as ABI1 and ABI2. This inhibition activates sucrose non-fermenting 1-related protein kinases (SnRK2s), which in turn phosphorylate ABA-responsive element-binding factors (ABFs), thereby regulating the expression of genes essential for AM symbiosis (Charpentier et al. 2014; Li et al. 2014). ABA also interacts with DELLA proteins, which act as repressors of gibberellin (GA) signaling and are essential for arbuscule formation. In *Medicagotruncatula* mutants lacking DELLA proteins, arbuscular development is impaired. However, overexpression of a dominant DELLA protein restored arbuscule formation, highlighting the crucial role of DELLA proteins in AM symbiosis (Charpentier et al. 2014; Floss et al. 2016). DELLA proteins interact with REQUIRED FOR ARBUSCULE DEVELOPMENT 1 (RAD1) and other GRAS factors that regulate transcription during AM symbiosis (Floss et al. 2016; Liao et al. 2018). ABA also enhances plant abiotic stress tolerance via AM symbiosis, regulating stress-related genes and maintaining shoot biomass and root hydraulic conductivity (Aroca et al. 2007). It also affects the expression of genes, such as *RAM1* and *DMI3*, which are essential for the establishment and maintenance of AM symbiosis (Gutjahr 2014). ABA interacts with ethylene to regulate AM formation. In tomato plants, the inhibition of ABA biosynthesis negatively impacts mycorrhization, whereas inhibition of ethylene synthesis in ABA-deficient plants increases mycorrhizal development. This dual mechanism suggests that ABA regulation of AM formation can be both ethylene dependent and independent (Herrera-Medina et al. 2007; de Los Santos et al. 2011; Martín-Rodríguez et al. 2011). In ABA-deficient tomato mutants, such as sitiens, overexpression of *ACC synthase* increases ethylene production, which negatively affects AM colonization. This effect can be reversed by inhibiting ethylene biosynthesis or perception and restoring mycorrhizal colonization (Fracetto et al. 2017). This makes ABA integral in enhancing plant resilience to environmental stresses, particularly through its role in AM symbiosis and water stress tolerance.

### ﻿Nitric Oxide (NO) and Karrikins (KARs): Modulating stress responses and AM symbiosis

In addition to the major phytohormones discussed, Nitric Oxide (NO) and karrikins (KARs) play critical roles in regulating plant-microbe interactions, particularly with AM. NO is vital for balancing plant immunity and symbiosis as it is involved in root development, defense responses, and communication with microbial partners. In legume-rhizobia interactions, for example, NO is crucial for root hair curling and nodule formation (Pande et al. 2021), and it also helps in the recognition of pathogen-associated molecular patterns (PAMPs), promoting both defense and symbiosis with AM (Khan et al. 2023). KARs derived from smoke interact with SL signaling pathways through the KAI2 receptor, influencing seedling morphology, root architecture, and AM colonization (Meng et al. 2022). KARs enhance root growth and nutrient uptake under abiotic stress, facilitating better symbiotic interactions, particularly with AM, by modulating auxin transport and promoting root hair development (Janssen and Snowden 2012). Overall, phytohormones, such as GAs, Ethylene, JA, ABA, and SLs, regulate AM symbiosis through complex, interlinked signaling networks. GAs and SLs mainly influence fungal colonization and arbuscule formation, with their effects varying depending on plant species and environmental factors. Ethylene and JA act as negative regulators, inhibiting fungal colonization and root development, whereas ABA and SLs modulate symbiotic efficiency and stress response. These hormones interact synergistically or antagonistically to balance the promotion of symbiosis with plant stress management. Understanding these interactions provides valuable insights into enhancing nutrient uptake, improving stress tolerance, and potentially boosting agricultural productivity.

## ﻿Role of AM in enhancing soil health and fertility

### ﻿Chemical enhancements and nutrient cycling

**Figure 3.**
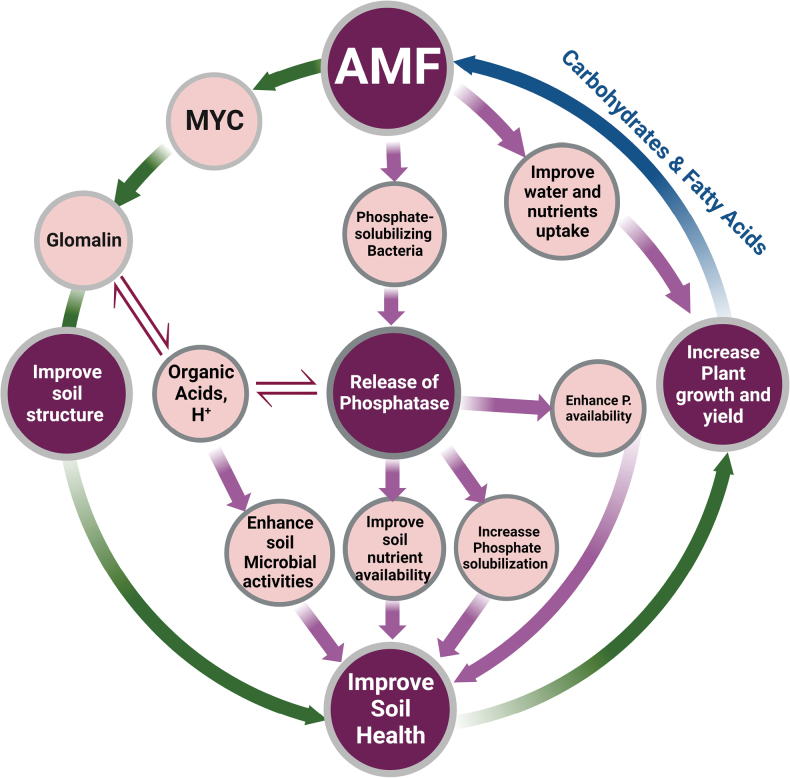
Role of AM in enhancing soil health: Contributions to soil chemical, physical, and biological properties, microbial diversity, and enzymatic activities for sustainable agriculture

AM plays a pivotal role in enhancing soil health and fertility by improving the chemical, physical, and biological properties of soils (Fig. 3). AM significantly contributes to soil chemical properties by improving the availability of essential nutrients, particularly phosphorus, which is often limited in many soils. AM achieves this through the production of organic acids and glomalin, a glycoprotein that chelates heavy metals and enhances nutrient bioavailability. Organic acids secreted by AM play a crucial role in the solubilization of phosphorus, making it more accessible to plants. Furthermore, these acids can regulate phosphatase activity, which is essential for the cycling of phosphorus, by mobilizing metal ions such as iron (Fe) and Zn, which are bound to phosphorus compounds (Li et al. 2019; Songachan 2023). In addition to improving phosphorus availability, AM contributes to soil carbon sequestration, stabilizing organic matter, and reducing atmospheric CO_2_ levels, which play a role in mitigating climate change (Songachan 2023). Thus, AM not only enhances nutrient cycling, but also contributes to broader environmental sustainability efforts.

### ﻿Physical properties and soil structure

Physically, AM enhances the soil structure by stabilizing soil macroaggregates. They bind soil particles via glomalin, a glycoprotein produced by fungi that improves water retention, increases soil aeration, and prevents erosion (Rillig et al. 2002). This is particularly beneficial under drought conditions, as AM hyphal networks enhance the soil’s water-holding capacity, allowing plants to maintain hydration and thrive even under water stress (Fall et al. 2022). Additionally, by stabilizing the soil structure and preventing soil erosion, AM helps maintain the integrity of the soil profile, which is crucial for supporting healthy plant growth and preventing degradation of agricultural lands (Agnihotri et al. 2022).

### ﻿Biological properties and microbial diversity

AM significantly enhances soil health and microbial diversity through interactions with a wide range of soil microorganisms. They contribute to the diversification of the rhizosphere and rhizosphere microbiomes by establishing symbiotic relationships with plant growth-promoting rhizobacteria (PGPR), such as *Pseudomonas* and *Bacillus* species, which enhance nutrient uptake and support root health (Sagar et al. 2021; Yu et al. 2022). These beneficial bacteria improve the soil microbial health, promote nutrient cycling, and foster a more balanced and resilient soil ecosystem. AM also promotes the growth of nitrogen-fixing bacteria, such as *Azospirillum* and *Rhizobium*, which convert atmospheric nitrogen into plant-accessible forms, thereby enhancing nitrogen availability in nitrogen-limited soils (Bauer et al. 2012; Ji et al. 2024). Furthermore, AM supports the proliferation of actinobacteria, such as *Streptomyces*, which are essential for organic matter decomposition and nitrogen fixation, thus enriching soil microbial diversity and improving soil health (Songachan 2023). In addition to these microbial interactions, AM improves nutrient availability by forming extensive hyphal networks that increase the surface area for nutrient absorption, particularly for phosphorus and nitrogen, in nutrient-limited soils (Cao et al. 2016; Frac et al. 2018). They also mitigate the negative effects of nanoparticles, such as Fe_3_O_4_NPs, on soil microbial communities, maintaining bacterial abundance and organic carbon levels in inoculated soils (Cao et al. 2016). Moreover, AM improves soil structure through the production of glomalin, a glycoprotein that helps bind soil particles, enhances water retention and aeration, and reduces soil erosion (Frac et al. 2018). In addition to plant nutrition, AM plays an integral role in broader ecological processes. They contribute to carbon sequestration by producing organic acids and glomalin, which stabilize soil aggregates and protect them against erosion. AM also influences the composition and activity of microbial communities involved in organic phosphorus mineralization, further supporting carbon storage. Studies have shown that AM diversity and network complexity enhance soil organic carbon (SOC) content, particularly in fallow lands, which underscores their role in mitigating climate change (Yang et al. 2022). Additionally, AM increases plant photosynthesis and soil respiration, leading to enhanced carbon storage in ecosystems, as demonstrated in coalfield soils, where AM treatment boosted carbon sequestration by 17.2% (Wang et al. 2016).

### ﻿Enzymatic contributions to soil fertility

AM also plays a crucial role in enhancing the enzymatic activities that drive nutrient cycling and organic matter decomposition. Phosphatases, which are produced by AM, mineralize organic phosphorus into bioavailable forms, which are particularly important in phosphorus-limited soils (Deng et al. 2024; Jin et al. 2024b). In addition, AM stimulates the activity of β-glucosidase, an enzyme that breaks down cellulose into glucose, which is utilized by soil microbes to fuel organic carbon accumulation (Ahmed et al. 2017). AM also enhances nitrogen metabolism by increasing the activities of enzymes, such as nitrate reductase and urease, which optimize nitrogen uptake and reduce nitrogen losses. For example, AM has been shown to promote the expression of nitrogen-fixing genes in maize soils, thereby increasing nitrogen availability for plants (Yin et al. 2009; Gou et al. 2024). Furthermore, AM enhances the activities of antioxidant enzymes, such as SOD and CAT, which help mitigate oxidative stress in plants and soil microbes by neutralizing ROS. These antioxidant activities are vital for supporting plant health, particularly under environmental stress conditions (Yang et al. 2015; Begum et al. 2019). Through these enzymatic processes, AM contributes to the stabilization of nutrient cycles and the promotion of organic matter accumulation, thereby enhancing soil fertility.

### ﻿AM and environmental sustainability

In addition to enhancing soil fertility, AM also plays an important role in promoting environmental sustainability. AM can help mitigate the toxic effects of heavy metals in soils through a process known as phytoremediation. Certain AM species, such as *Claroideoglomusetunicatum* and *Rhizophagusintraradices*, have been shown to reduce the transport of heavy metals, such as arsenic and molybdenum, to plant shoots, thus alleviating metal-induced phytotoxicity (Zhang et al. 2023; Ahmed et al. 2024a). This makes AM particularly valuable in contaminated soils, where it helps plants cope with metal toxicity. Furthermore, the combined use of AM and biochar has been found to improve soil structure, increase soil pH, and enhance the availability of organic carbon and phosphorus, thereby boosting soil fertility (Vogel-Mikuš et al. 2006; Ahmed et al. 2024c). These fungi not only contribute to the sustainability of agricultural systems but also play a significant role in mitigating climate change by stabilizing soil organic matter and enhancing carbon sequestration. Their potential to improve soil health, combined with their ability to support environmental sustainability, makes AM a crucial component of sustainable agricultural practices and soil-management strategies.

## ﻿Role of AM in enhancing nutrient availability and uptake

AM plays a crucial role in improving nutrient availability and plant growth, particularly in nutrient-poor soil. By forming symbiotic relationships with plant roots, AM extend their hyphal networks into the soil, increasing the surface area for nutrient absorption. This enhances the plant’s ability to acquire essential nutrients, reduces reliance on synthetic fertilizers, and supports sustainable agriculture. In fact, AM interactions can reduce the need for synthetic phosphorus fertilizers by up to 90% (Ebbisa 2022; jha and Songachan 2023; Wu et al. 2024). Phosphorus is often limited in soils, particularly in phosphorus-deficient environments. AM enhances phosphorus uptake by extending hyphal networks beyond root depletion zones and secreting phosphatases that hydrolyze organic phosphorus, making it available to plants. Qi et al. (2022) showed that AM, such as *Rhizophagusintraradices* can significantly improve phosphorus acquisition under insoluble conditions. Additionally, AM forms symbiotic relationships with phosphate-solubilizing bacteria (PSB), which produce organic acids that further solubilize the mineral phosphorus. Co-inoculation with AM and PSB increased phosphorus bioavailability and plant growth (El Maaloum et al. 2020). Additionally, AM regulates the expression of phosphorus transporter genes, such as *SlPT3*, in tomatoes to improve phosphorus uptake efficiency (Rui et al. 2023). In addition to phosphorus, nitrogen is also essential for plant growth. AM improves nitrogen use efficiency (NUE) by accessing nitrogen sources outside the root zone and transferring it through common mycorrhizal networks (CMNs) (Luo et al. 2023; Muneer et al. 2023). This improves nitrogen distribution and enhances plant growth in nitrogen-limited environments. For example, in maize, AM upregulates genes such as *ZmAMT3;1*, which boost ammonium transport (Hui et al. 2022). Furthermore, AM improves NUE under elevated CO_2_ conditions, thereby mitigating nitrogen loss and reducing environmental impacts (Panneerselvam et al. 2019).

AM also enhances potassium uptake, especially in acidic or saline soils where potassium is less available. They work with potassium-solubilizing microorganisms (KSMs) to release potassium from mineral forms, thereby improving their accessibility (Olaniyan et al. 2022; Singh et al. 2022). Additionally, AM regulates potassium transporter genes such as *LbHAK* in *Lyciumbarbarum* to increase potassium uptake (Zhang et al. 2024b). In saline-alkaline soils, AM enhances resilience by improving the K^+^/Na^+^ ratio, which helps plants tolerate salt stress (Dong et al. 2023; Nawaz et al. 2023). Similarly, AM promotes Mg uptake through transport mechanisms that allow plants to access this important nutrient, which is vital for chlorophyll synthesis and plant stress tolerance (Zai et al. 2014; Ahmed et al. 2023). Furthermore, AM enhances sulfur uptake by inducing the expression of sulfur transporter genes, such as *LjSultr1;2* in *Lotusjaponicus* (Casieri et al. 2012) and stimulating sulfur-solubilizing bacteria in the rhizosphere, thereby improving sulfur bioavailability (Nadeem et al. 2023). These interactions enhance macronutrient uptake, reduce fertilizer dependency, and support sustainable agricultural practices.

In addition to macronutrients, AM also contributes significantly to the uptake of several micronutrients that are essential for plant growth and development. For instance, AM enhances zinc uptake by upregulating zinc transporter genes, such as *MtZIP14* in *Medicagotruncatula* and *BoZIP1* in *Brassicaoleracea* (broccoli), and they may also release organic acids to improve zinc solubility in the rhizosphere (Watts-Williams et al. 2023; Arifuzzaman et al. 2024). In iron-deficient soils, AM enhances iron uptake by activating iron transporter genes such as *MsFRO1* and *MsIRT1* in *Medicagosativa* (alfalfa) and by secreting siderophores that improve the solubility and bioavailability of iron in the rhizosphere (Rahman et al. 2020). This interaction not only enhances iron acquisition but also contributes to improved plant biomass, chlorophyll content, and photosynthetic efficiency. Additionally, AM inoculation elevated the levels of S-metabolites (e.g., glutathione and cysteine), providing antioxidant protection against oxidative stress induced by iron deficiency, thereby promoting better plant health and growth under Fe-deficient conditions (Rajapitamahuni et al. 2023). Additionally, AM enhances Cu uptake by immobilizing excess Cu in Cu-deficient or contaminated soils, thereby reducing toxicity. In poplars, *Rhizophagusirregularis* and *Paraglomuslaccatum* influenced copper translocation and plant resilience with species-specific effects (Soltangheisi et al. 2024). Additionally, *Rhizophagusirregularis* regulates copper homeostasis via *RiCRD1*, which plays a role in copper detoxification and transfer at the symbiotic interface (Gómez-Gallego et al. 2024). Together, these studies highlight AM ’s role of AM in Cu uptake and toxicity mitigation.

AM enhances boron (B) acquisition in plants by regulating boron transporter genes, such as *BOR1*, facilitating boron uptake under stress. In *Camelliaoleifera*, AM inoculation increased boron content and antioxidant enzyme activity, improving resistance to boron deficiency, although B deficiency itself reduced AM root colonization (Liu et al. 2023). Similarly, the *NIP5;1* gene in *Arabidopsisthaliana* enhances boron uptake under B limitation (Takano et al. 2006). In tomato plants exposed to high boron stress, AM inoculation mitigated excessive boron uptake, improved growth parameters, and reduced leaf boron concentrations, offering a potential strategy for managing boron toxicity in contaminated soils (Turhan 2021). AM alleviates manganese (Mn) toxicity in plants by modifying its solubility and enhancing antioxidative responses. In *Rhuschinensis*, AM inoculation with *Funneliformismosseae* increased Mn accumulation in the roots while sequestering it in less toxic forms in the cell wall, reducing phytotoxicity (Pan et al. 2023). Similarly, in soybean, AM inoculation improved Mn translocation and boosted antioxidative enzyme activity under excess Zn, supporting better Mn management and plant resilience (Ibiang et al. 2017).

AM enhances (Mo) acquisition by regulating Mo transporter activity, which is crucial for nitrogenase function in nodules. For instance, *Medicagotruncatula MtMOT1.2* facilitates Mo transport to the cytosol, supporting nitrogen fixation (Gil-Díez et al. 2019). Additionally, AM helps plants to manage Mo toxicity by enhancing uptake at low concentrations and mitigating oxidative stress at high levels, thereby improving plant growth and nitrogen fixation efficiency (Shi et al. 2018; Yang et al. 2024). They also aid in silicon solubilization and improve plant resistance to drought, salinity, and pathogen stress, thus playing a vital role in stress tolerance (Garg and Bhandari 2016). AM helps manage chloride (Cl^−^) homeostasis under saline conditions by promoting its sequestration in vacuoles, reducing chloride toxicity, and supporting plant growth. For example, *Funneliformismosseae* inoculation in *Populussimonii × P.nigra* under saline-alkali stress improved chloride distribution, enhanced plant growth, and regulated Na^+^ and Cl^−^ homeostasis. In addition, AM boosts water and nutrient uptake, further increasing plant tolerance to salt stress (Evelin et al. 2019; Dong et al. 2023). This enhanced nutrient uptake, facilitated by AM, is further supported by the regulation of plant gene expression. AM upregulates nutrient transporter genes to optimize the absorption of essential micronutrients, thereby contributing to plant growth, stress resilience, and overall health.

## ﻿Mechanisms of abiotic stress tolerance and crop yield and quality improvements mediated by AM

**Figure 4.**
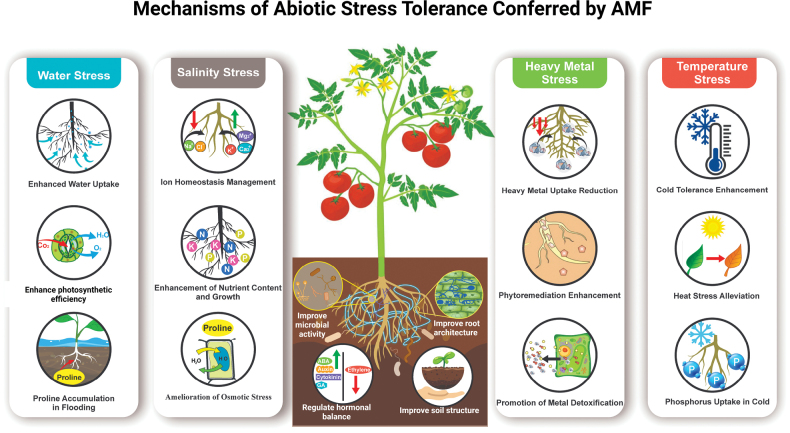
Schematic overview of AM -mediated key strategies to confer resistance to various abiotic stresses in host plants.

AM plays a significant role in improving plant tolerance to various abiotic stresses, including drought, salinity, heavy metals, and extreme temperatures. The mechanisms by which AM enhances abiotic stress tolerance are multifaceted, involving physiological, biochemical, and molecular changes in plants (Fig. 4).

### ﻿Modulation of antioxidant defense systems

One of the primary mechanisms through which AM helps plants cope with abiotic stress is by enhancing their antioxidant defense systems. Abiotic stresses such as drought, salinity, and metal toxicity lead to the overproduction of ROS, which causes oxidative damage to plant cells (Zulfiqar et al. 2022; Hayat et al. 2023). AM inoculation stimulates the production of antioxidants, such as superoxide dismutase (SOD), catalase (CAT), and peroxidase (POD), which neutralize ROS and reduce oxidative stress (Begum et al. 2019). At the molecular level, AM enhanced the expression of genes associated with ROS detoxification. For example, genes encoding SOD and CAT are upregulated in mycorrhizal plants under stress conditions, enabling them to scavenge excess ROS more efficiently (Yang et al. 2015; Arifuzzaman et al. 2024). Furthermore, AM has been shown to increase the synthesis of glutathione, a key antioxidant involved in maintaining cellular redox balance. Increased antioxidant capacity not only reduces oxidative damage but also promotes overall plant health and stress resilience.

### ﻿Improved water use efficiency and abiotic tolerance

AM contributes significantly to improving drought tolerance by enhancing water-use efficiency. Mycorrhizal association leads to changes in root architecture, including the development of finer and more extensive root systems, which improve water uptake from the soil (Zhang et al. 2019a; Mitra et al. 2021). AM also enhances the plant’s ability to maintain water balance under drought conditions by modulating the production of ABA, a key plant hormone involved in the drought response. AM influences ABA biosynthesis in the roots, which triggers stomatal closure, thereby reducing water loss through transpiration during drought stress (Ahmed et al. 2019; Begum et al. 2019). Additionally, AM improves the ability of plants to accumulate osmolytes, such as proline, trehalose, and melatonin, which helps to maintain cellular turgor and stabilize proteins under water deficit conditions (Ghani et al. 2022; Ahmad et al. 2024). These molecular and physiological changes significantly increase the ability of plants to withstand drought stress and improve water-use efficiency.

### ﻿AM mediated enhanced soil detoxification

AM plays a crucial role in detoxifying soils contaminated with heavy metals and offers a sustainable solution for polluted environments. Species such as *Rhizophagusirregularis* and *Claroideoglomusetunicatum* sequester metals such as cadmium (Cd), lead (Pb), arsenic (As), and chromium (Cr) in their hyphae, reducing their bioavailability to plants (Riaz et al. 2021; Kaur et al. 2022; Ahmed et al. 2024a). This process is further supported by the production of metallothioneins, glutathione, and phytochelatins, which form stable metal complexes to alleviate metal toxicity. For example, *Gigasporamargarita* produces metallothionein-like proteins that chelate Cd and Cu, reducing toxicity to both fungal and plant partners (Lanfranco et al. 2002; Miransari 2017; Riaz et al. 2021). Additionally, species such as *Diversisporaepigaea* immobilize heavy metals, such as Pb and Cr, through the production of extracellular polymeric substances (EPS), which bind and sequester metal ions and enhance soil detoxification (An et al. 2022). AM also alters rhizosphere chemistry by releasing organic acids such as citric and oxalic acid, which chelate heavy metals and reduce their solubility and bioavailability (Aguilera et al. 2015). For instance, *Acaulosporalaevis* acidifies the soil, converting metals, such as Pb and Cu, into less bioavailable forms. Fungi such as *Aspergillusniger* produce organic acids such as gluconic acid, which solubilize and immobilize metals, demonstrating their potential for remediating contaminated soils (Ren et al. 2009; Giese et al. 2024). Furthermore, AM ‘s interaction of AM with metal-resistant bacteria enhances their ability to detoxify metals through efflux pumps and stress response mechanisms (Zadel et al. 2022). Together, these mechanisms make AM an effective tool to mitigate soil contamination, improve soil health, and support environmental sustainability.

### ﻿Crop yield improvement mediated by AM

The application of AM has resulted in significant improvements in crop yield across various agricultural systems and crops. For instance, in maize, AM inoculation via seed coating combined with phosphorus fertilizer resulted in a 30% increase in grain yield, while reducing the need for phosphorus fertilizer by 50% (Marwanto et al. 2024). Similarly, in wheat, AM inoculation under drought conditions led to a 28.5% increase in grain yield, showing AM ’s potential of AM to enhance water-use efficiency and nutrient uptake, especially under water-limited conditions (Duan et al. 2024). AM ’s positive impact of AM is also evident in rice, where the combination of AM and nitrogen fertilizer led to substantial improvements in yield components, including a 30% increase in 1000-grain weight (Mulyadi and Jiang 2023). Additionally, a meta-analysis of cereal crops, including maize, wheat, and rice, found an average yield increase of 16% following AM inoculation, although this effect varied depending on the crop type and environmental conditions (Zhang et al. 2019b). In potatoes, AM inoculation resulted in a 9.5% increase in marketable yield, highlighting the economic benefits of AM application in large-scale agriculture (Hijri 2016). In cotton, AM applications resulted in a 28.54% increase in seed cotton yield, along with improved phosphorus acquisition and fiber quality (Gao et al. 2020). These findings illustrate the effectiveness of AM in boosting crop yield under diverse environmental conditions.

### ﻿AM effect on crop quality attributes

In addition to improving crop yields, AM inoculation has also been shown to enhance various quality attributes of crops, thereby improving their nutritional and market value. In fruits such as strawberries, AM inoculation increased key physicochemical properties, such as pH, titratable acidity, and soluble solids content, contributing to better taste, longer shelf life, and overall marketability (Cordeiro et al. 2019). Moreover, AM and selenium (Se) biofortification in garlic and onion resulted in an improved biochemical composition, with increases in monosaccharides, selenium content, ascorbic acid, and flavonoids, along with enhanced mineral composition (Golubkina et al. 2020). AM inoculation has also been linked to a higher soluble sugar content in fruits, which directly influences sweetness and flavor. For example, tomatoes from mycorrhizal plants exhibited higher BRIX values, indicating enhanced sweetness and improved taste quality compared with non-mycorrhizal plants (Schubert et al. 2020). Additionally, mycorrhization has been shown to increase the abundance of free amino acids in fruits, particularly asparagine and glutamine, thereby improving their nutritional profiles and potential health benefits (Salvioli et al. 2012). AM has also been associated with higher carotenoid levels in fruits such as tomatoes, which contribute to antioxidant properties and enhance both the color and nutritional value of the produce (Schubert et al. 2020; Chachar et al. 2024). Furthermore, in cotton, AM symbiosis improves fiber quality and enhances traits such as fiber length, strength, and fineness, thus underscoring the multifaceted benefits of AM on crop quality (Gao et al. 2020). AM application boosts crop yield by enhancing nutrient uptake and growth while also improving quality traits such as flavor, sweetness, antioxidants, and nutrition, making it a valuable tool for both productivity and crop quality enhancement.

### ﻿Impact of environmental stressors on AM colonization and functionality

The impact of environmental stressors on AM colonization and functionality is often overlooked, despite AM ‘s role in enhancing plant resilience to abiotic stresses. Drought, salinity, heavy metals, temperature fluctuations, and soil conditions significantly affect AM colonization, community composition, and functionality, which are crucial for optimizing their effectiveness in agriculture. For instance, drought conditions hinder AM growth and reproduction, leading to reduced spore germination, hyphal elongation, and formation of extraradical mycelia, which limits nutrient transfer (Bahadur et al. 2019; Albracht et al. 2023). Moreover, drought stress can shift AM community composition, reducing species diversity, although α-diversity may remain stable in some cases (Albracht et al. 2023). These changes in AM dynamics can impair their ability to enhance plant drought tolerance, creating a feedback loop in which less effective AM fails to support plant health under stress (Bahadur et al. 2019; Jajoo and Mathur 2021). Similarly, high salinity inhibits AM colonization by affecting spore germination and hyphal growth due to the osmotic stress caused by NaCl (Ruiz-Lozano et al. 2012; Klinsukon et al. 2021). However, some AM species are more salt-tolerant, potentially helping plants mitigate the negative effects of salinity through improved nutrient uptake and osmotic regulation (Ruiz-Lozano et al. 2012; Klinsukon et al. 2021).

Heavy metals also pose significant challenges to AM, as they can impair spore germination and hyphal growth and disrupt the symbiotic relationship between AM and host plants (Hildebrandt et al. 2007; Menge and Menge 2023). Despite these challenges, some AM species have shown potential in alleviating heavy metal stress in plants, either by sequestering metals or enhancing plant tolerance through physiological adjustments (Menge and Menge 2023; Ahmed et al. 2024a). Temperature fluctuations further complicate AM functionality, with high temperatures reducing colonization rates and impairing symbiotic relationships (Begum et al. 2019; Jian et al. 2024). Conversely, low temperatures may enhance certain aspects of AM functionality, such as nutrient uptake efficiency, although prolonged exposure can lead to metabolic slowdowns and reduced fungal activity (Begum et al. 2019; Jian et al. 2024). Edaphic factors such as soil pH, nutrient availability, and moisture levels also influence AM dynamics. Highly acidic or alkaline soils can reduce fungal diversity and colonization rates (Menge and Menge 2023), whereas nutrient-poor soils tend to favor certain AM species that are better adapted to such conditions. Soil moisture also plays a crucial role; moderate moisture supports AM growth, but excessive waterlogging can hinder fungal development due to anaerobic conditions (Bahadur et al. 2019; Jajoo and Mathur 2021). Thus, understanding how environmental stressors affect AM is essential for optimizing its role in agriculture, particularly in mitigating the negative impacts of abiotic stresses on plant health and productivity.

## ﻿Challenges in optimizing AM inoculation techniques for enhancing plant resilience and productivity

AM is a vital component of sustainable agriculture, offering numerous benefits to plant health and productivity under various environmental stressors. However, optimizing AM inoculation techniques is challenging due to factors such as species diversity, host plant specificity, environmental variability, and practical scalability (Kivlin et al. 2011; Lee et al. 2013).

**Table 1.** Effects of AM inoculation on crop yield and quality improvement.

Crop	Treatment	Yield Increase	Key Nutrient/Quality Improvement	Reference
Maize	AM + Phosphorus Fertilizer	30% increase in grain yield	50% reduction in P fertilizer use; improved P uptake	(Marwanto et al. 2024)
Wheat	AM (Drought)	28.5% increase in grain yield	Improved water use efficiency; better nutrient uptake	(Duan et al. 2024)
Rice	AM + Nitrogen	67.44% increase in panicle number	30.70% increase in 1000-grain weight; improved N uptake	(Mulyadi and Jiang 2023)
Cotton	AM inoculation (*Rhizophagusirregularis CD1*)	28.54% increase in seed cotton yield	Increased P acquisition (13.65%–43.27%); enhanced photosynthesis, plant growth, boll number, and fiber maturity	(Marwanto et al. 2024)
Garlic/Onion	AM + Selenium	Highest bulb yield	Increased monosaccharides, selenium, flavonoids, and minerals (P, K, Ca, Mg, B, Fe, Zn)	(Golubkina et al. 2020)
Tomato	AM inoculation (*Rhizophagusirregularis*) in hydroponics	No significant effect on yield, maintained under low phosphate conditions	Increased BRIX values in red fruits; higher carotenoid levels; free amino acids up to four times higher	(Schubert et al. 2020)
Wheat	Mycorrhizal Inoculation (Consortia)	Grain yield increased by 21.2%	Protein, Zn, Fe, P, K, and organic carbon levels were significantly improved.	(Akbar et al. 2023)
Maize	AM Inoculation (4 species mix, seed treatment)	Yield increases with AM rate 20 ml ha^-1^ applied with P fertilization	Growth-promoting effects observed, no specific nutrient improvement	(Pedroso et al. 2022)
Soybeans	AM Inoculation (4 species mix, seed treatment)	Yield increase when AM rate 20 ml ha^-1^ applied with P fertilization	No specific nutrient improvement mentioned but growth-promoting effects observed	(Pedroso et al. 2022)
Tef	AM inoculation (mixture of 4 AM species)	Increased root colonization and growth	Root and shoot morphology improved; Increased root length, shoot biomass, plant height, and panicle length. Nutrient uptake enhanced, leading to improved performance.	(Gebremeskel et al. 2024)
Tomato	AM Inoculation (5 species: *A.morrowiae*, *P.occultum*, *F.mosseae*, *R.clarus*, *R.intraradices*)	Increase in shoot dry weight and yield	Enhanced antioxidant enzyme activity, improved photosynthetic pigments, reduced levels of ROS like MDA and H_2_O_2_, and increased concentrations of essential minerals such as K, Ca, Mg, and Fe.	(Alam et al. 2023)
Two-rowed Barley	AM inoculation with different fertilization regimes	22% higher seed yield compared to untreated plots	AM inoculation improved plant height, LAI, nitrogen and phosphorus uptake, and their utilization indices.	(Beslemes et al. 2023)
Maize	AM application vs. No AM application (CK) in sandy and saline–alkali soils	25.77% increase in sandy soil; 18.63% increase in saline–alkali soil	Enhanced root growth, yield, grain quality, and improved soil nutrients (N, P, K). Increased soil microbial diversity and richness, with notable changes in microbial communities.	(Fan et al. 2024)
Wheat	AM (*Rhizophagusintraradices*) + Biotol (PGPR) Inoculation	Seed yield increased (Sakha 93: 1.99 t/ha; Gemmeza 9: 1.71 t/ha)	Improved yield and salt tolerance under reduced NPK and high salinity conditions; higher proline and salicylic acid levels	(Aboul-Nasr et al. 2016)
Rice	AM +PGPR (30 ml/L)	Number of panicles per plant increased; Grain weight per plant increased	Improved number of panicles, grain weight, and overall production components compared to control	(Aswad et al. 2023)
Potato	AM inoculation (*Rhizophagusirregularis*)	9.5% increase in marketable yield (3.9 tons/ha)	Significant increase in marketable yield; profitable, with a 0.67-ton/ha increase in 79% of trials	(Hijri 2016)

### ﻿AM species diversity

The diversity of AM species, such as *Rhizophagusintraradices*, *Funneliformismosseae*, and *Claroideoglomusetunicatum*, influence plant stress tolerance and nutrient acquisition. However, their effectiveness varies across different ecosystems. For example, *R.intraradices* enhance lead tolerance in soybeans (Adeyemi et al. 2021; Chauhan et al. 2022), whereas *C.etunicatum* and *R.intraradices* improve the biomass and nutrient uptake in other plants. However, these species perform differently under various environmental conditions. *R.intraradices* show better molybdenum tolerance than *C.etunicatum* (Zhang et al. 2023), and *Rhizophagusirregularis* and *Claroideoglomusclaroideum* enhance nutrient uptake and reduce fertilizer reliance (Jia et al. 2023), but these benefits are not universal, as the same species may exhibit different performances across ecosystems or crop types due to specific environmental conditions and host plant preferences.

### ﻿Host plant specificity and compatibility

AM species exhibit high host specificity, which influences their compatibility with different plants. Some crops, such as wheat and maize, respond well to AM inoculation, whereas others, such as legumes and rice, show limited benefits due to differences in root architecture and symbiotic preferences (Yang et al. 2012; Selvakumar et al. 2018). For instance, *R.intraradices* enhances water stress tolerance and aphid defense in tomato plants, whereas other species such as *Funneliformismosseae* are less effective (Volpe et al. 2018). Environmental factors, such as soil properties, moisture, and temperature, also affect compatibility, complicating the generalization of AM applications across crops (Yang et al. 2012; d’Entremont and Kivlin 2023). Additionally, invasive species, such as *Acaciadealbata* can disrupt native plant-AM interactions, altering soil properties and reducing biodiversity (Guisande-Collazo et al. 2022). Understanding these complexities is crucial for optimizing AM-based agricultural strategies.

### ﻿Optimization of application timing

The timing of AM inoculation significantly affected its effectiveness. Early-stage inoculation generally yielded better outcomes. For example, the inoculation of lettuce at the sowing and transplanting stages produced similar growth responses, indicating that AM infection can be effective at multiple plant development stages (Wee et al. 2010). In chrysanthemums, inoculation at transplanting enhanced rooting, growth, and flowering time, with the best results observed when applied directly to cuttings (Attia and Rawia 2005). Similarly, pre-transplantation inoculation of ginseng enhanced growth and productivity (Cho et al. 2009). Early inoculation in perilla improved leaf area, shoot length, and root weight (Sohn et al. 2010), whereas in maize-soybean intercropping, early inoculation altered the AM community composition and improved yields (Wang et al. 2022b), alleviating salt stress during nursery establishment (Balliu et al. 2015). These findings emphasize the importance of early-stage AM inoculation for optimizing plant growth and stress resilience.

### ﻿Interplay of AM with environmental and soil factors

Environmental factors, such as soil type, nutrient availability, and biotic interactions, significantly affect AM inoculation outcomes. AM species such as *Rhizophagusirregularis* and *Claroideoglomusclaroideum* enhance growth in *Imperatacylindrica* in copper tailings (Jia et al. 2023), while elevated CO_2_ boosts maize yield and defends against species such as *Funneliformiscaledonium* (Wang et al. 2021a). AM inoculation also improved saffron yield under harsh conditions (Caser et al. 2018) and enhanced drought resistance in *Alhagisparsifolia* (Aili et al. 2023). In maize, the effects of AM vary with soil microbial communities (Dias et al. 2018), and olive trees benefit from AM under saline conditions, although pathogen resistance depends on environmental stress (Kapulnik et al. 2010). These results highlight the importance of soil and environmental factors in determining the effectiveness of AM.

### ﻿Application methods

Different AM inoculation methods have been explored to optimize biotic and abiotic stress management. Studies have highlighted the effectiveness of various application techniques including direct soil inoculation, pre-inoculation of seeds or seedlings, and foliar application of AM spore suspensions (Selvakumar et al. 2018; Xiao et al. 2022). For example, direct soil inoculation with AM significantly reduced root rot in *Panaxnotoginseng* and improved its disease resistance (Ren et al. 2023). Additionally, using root-baiting methods, where plant roots are attracted to AM inoculum, can effectively improve plant health (Knerr et al. 2018). Combining AM inoculation with biochar application has also been shown to increase root colonization, improve soil properties, and enhance plant growth and drought tolerance (Jabborova et al. 2022; Ahmed et al. 2024c). These findings demonstrate that the application techniques significantly influence the potential of AM to promote plant growth and productivity.

### ﻿Potential downsides of AM use

Despite its benefits, the widespread use of AM in agriculture has potential disadvantages. First, the introduction of AM can alter plant community structure, potentially reducing biodiversity by favoring some species over others (Jin et al. 2017). Furthermore, AM may compete with other soil microorganisms for root exudates, disrupting other beneficial plant-microbe interactions, such as rhizobia and PGPRs (Hodge and Fitter 2013; Vishwakarma et al. 2017). AM applications may also be less effective in nutrient-rich soils where plants do not rely heavily on symbiosis (Touré et al. 2023). In some cases, AM can become parasitic, extracting more nutrients from the host plant than they provide, potentially reducing plant growth and crop yield (Reynolds et al. 2005; Johnson 2010). These potential drawbacks highlight the need for careful management and monitoring of AM applications to ensure that their benefits outweigh their risks.

### ﻿Challenges in AM application across diverse crops, soil conditions, and climates

Despite the significant benefits that AM offers in terms of nutrient uptake, stress tolerance, and soil structure enhancement, its application in agriculture faces several challenges. The effectiveness of AM inoculation varies considerably depending on crop species, soil conditions, and environmental factors. While crops such as wheat and maize consistently benefit from AM, other crops such as legumes and rice may show limited or inconsistent responses (Yang et al. 2012; Selvakumar et al. 2018). Furthermore, even within the same crop species, AM performance can vary depending on the environmental conditions. For example, *Rhizophagusintraradices* enhance drought tolerance in some plants, but not in others (Volpe et al. 2018). Soil properties, such as pH, nutrient levels, and temperature, also affect AM colonization, making it necessary to tailor AM application strategies to specific environmental conditions (Kivlin et al. 2011). In particular, high phosphorus levels can inhibit AM colonization, whereas lower soil organic carbon and microbial biomass levels can make AM more critical for plant growth (Lutz et al. 2023). Agricultural practices, such as tillage, can further disrupt AM networks, thereby reducing their effectiveness. In contrast, practices such as reduced tillage and cover cropping can promote AM colonization (Schaefer et al. 2021).

In addition, crop species differ in their ability to form effective AM symbioses. For example, potatoes respond more consistently to AM than wheat, and selective breeding for yield-related traits may reduce the responsiveness of modern cultivars (Tian et al. 2017; Mitra et al. 2023; Watts et al. 2023; Zhang et al. 2024b). The quality of commercially available AM inoculants can also vary, with some products containing non-viable propagules further complicating their application (Watts et al. 2023). To address these issues, it is essential to establish quality control measures for the inoculants. Environmental factors, such as temperature and water availability, also affect the effectiveness of AM. Although higher temperatures may increase mycorrhizal abundance in some cases, they can reduce colonization in others (Aguilera et al. 2022). Moreover, promoting native AM communities through sustainable practices such as crop diversification and organic farming may reduce the need for external inoculants (Lutz et al. 2023). Nonetheless, AM inoculation should be tailored to specific crops, soils, and microbial communities to ensure its success (Watts et al. 2023). Finally, ongoing field trials and research are essential to address the remaining uncertainties surrounding AM applications, helping refine strategies and improve their practical use in diverse agricultural systems.

## ﻿Conclusion and future perspectives

AM plays a crucial role in enhancing plant resilience to abiotic stresses such as drought, salinity, and temperature extremes. AM significantly contributes to plant health and stress tolerance by improving nutrient uptake, water absorption, and activating stress-related pathways. Additionally, AM enhances soil health through improved structure, organic matter content, and microbial diversity, which further support nutrient cycling and soil fertility. However, their effectiveness is influenced by factors such as the AM species, plant genotype, and environmental conditions, underscoring the need for optimized inoculation strategies. Future research should focus on elucidating the molecular mechanisms behind AM-mediated stress tolerance, exploring synergies with other agricultural practices, such as plant breeding, and assessing their long-term ecological impact on soil biodiversity. Scaling up AM inoculant production, improving compatibility with diverse crops, and addressing the variability in field performance are critical challenges. Advances in biotechnological tools, such as genetic modifications and bioformulations, offer promising avenues for enhancing the efficacy of AM. Additionally, precision agriculture technologies can optimize AM use and improve resource efficiency and crop resilience across different agroecological settings. With continued research, better inoculation techniques, and broader adoption, AM can significantly contribute to climate change mitigation, sustainable agriculture, and food security. Interdisciplinary collaboration, policy support, and farmer education are essential to fully integrate AM into global agricultural systems.

## ﻿Additional information

### Adherence to national and international regulations

All the fungal strains used in this study have been legally obtained, respecting the Convention on Biological Diversity (Rio Convention).

### Author ORCIDs

Nazir Ahmed https://orcid.org/0000-0001-5830-7038

Muzafaruddin Chachar https://orcid.org/0000-0003-2123-2110

Sadaruddin Chachar https://orcid.org/0000-0002-9714-7775

### Data availability

All of the data that support the findings of this study are available in the main text.
